# Detachment and successive re-attachment of multiple, reversibly-binding tethers result in irreversible bacterial adhesion to surfaces

**DOI:** 10.1038/s41598-017-04703-8

**Published:** 2017-06-29

**Authors:** Jelmer Sjollema, Henny C. van der Mei, Connie L. Hall, Brandon W. Peterson, Joop de Vries, Lei Song, Ed D. de Jong, Henk J. Busscher, Jan J. T. M. Swartjes

**Affiliations:** 1 University of Groningen and University Medical Center Groningen, Department of Biomedical Engineering, Antonius Deusinglaan 1, 9713 AV Groningen, The Netherlands; 2 Department of Biomedical Engineering, The College of New Jersey, Armstong Hall, Room 181, P. O. Box 7718, The College of New Jersey, Ewing, NJ 08628 USA

## Abstract

Bacterial adhesion to surfaces occurs ubiquitously and is initially reversible, though becoming more irreversible within minutes after first contact with a surface. We here demonstrate for eight bacterial strains comprising four species, that bacteria adhere irreversibly to surfaces through multiple, reversibly-binding tethers that detach and successively re-attach, but not collectively detach to cause detachment of an entire bacterium. Arguments build on combining analyses of confined Brownian-motion of bacteria adhering to glass and their AFM force-distance curves and include the following observations: (1) force-distance curves showed detachment events indicative of multiple binding tethers, (2) vibration amplitudes of adhering bacteria parallel to a surface decreased with increasing adhesion-forces acting perpendicular to the surface, (3) nanoscopic displacements of bacteria with relatively long autocorrelation times up to several seconds, in absence of microscopic displacement, (4) increases in Mean-Squared-Displacement over prolonged time periods according to t^α^ with 0 < α ≪ 1, indicative of confined displacement. Analysis of simulated position-maps of adhering particles using a new, *in silico* model confirmed that adhesion to surfaces is irreversible through detachment and successive re-attachment of reversibly-binding tethers. This makes bacterial adhesion mechanistically comparable with the irreversible adsorption of high-molecular-weight proteins to surfaces, mediated by multiple, reversibly-binding molecular segments.

## Introduction

Bacteria prefer to live in surface-associated communities called biofilms, rather than to live their life planktonically in suspension, because the biofilm-mode of growth offers several advantages, including amongst others, protection against environmental challenges^1^. The complexity of bacterial cell surfaces, often possessing a layer of extracellular polymeric substances (EPS) and a variety of differently termed proteinaceous surface appendages, such as fibrils or fimbriae^2^, has made it impossible to define a generally valid mechanism by which bacteria adhere to surfaces. Bacteria initially adhere reversibly to a surface, but within several minutes after first contact, the forces through which bacteria adhere become stronger and adhesion more irreversible. This early transition to irreversible adhesion occurs due to physico-chemical processes such as (1) the progressive removal of interfacial water, (2) bacteria seeking to expose their most adhesive sites towards a surface and (3) macromolecular re-arrangements in the tethers through which they bind^3^. Apart from these early physico-chemical processes, adhering bacteria can actively produce EPS to anchor themselves more firmly to a surface, depending on their genetic ability to do so, nutrient availability and temperature^4, 5^. Although bacteria are known to adhere irreversibly within several minutes, the requirement of reversibility is often ignored in the application of surface thermodynamic^6^ and Langmuir-type^7^ analyses of bacterial adhesion. Yet, not seldom these approaches have their merits^8, 9^. Unfavorable, positive values for the interfacial free energies of bacterial adhesion derived from measured contact angles with liquids and surface thermodynamic approaches for instance, have been demonstrated to coincide with a greater reversibility than in cases of negative interfacial free energies of adhesion that are favorable for adhesion^8^. At the same time, surface thermodynamics of bacterial detachment and DLVO analyses have indicated that in order to detach from a surface, adhering bacteria have to escape an energy well with a depth of up to several tens of 10 kT^10, 11^, which is impossible with their thermal, Brownian-motion energy of 1 kT. This poses the question up to what extent, from a physico-chemical perspective, bacterial adhesion truly is an irreversible phenomenon.

Bacteria adhere to surfaces through viscoelastic rather than through rigid bonds. The elasticity and length of the bond are reflected in Brownian-motion induced, random motion of the adhering bacteria that is confined by the bond characteristics themselves, similar as observed for particles tethered to a surface through DNA or RNA strands^12, 13^ or for abiotic latex particles, tethered through polymer extrusions^14^. Here we hypothesize that multiple, reversibly-binding bacterial tethers detach and successively re-attach, but never detach all at the same time, resulting in irreversible bacterial adhesion. In order to prove that bacteria adhere to substratum surfaces through multiple, detaching and successively attaching tethers, we combine various analyses of the confined Brownian-motion of adhering bacteria^15^ and their adhesion forces as obtained using bacterial probe Atomic Force Microscopy (AFM)^16^. Conclusions are confirmed by analysis of simulated position-maps of adhering particles using a new, *in silico* model, that simulates Brownian-motion of a particle adhering to a substratum surface by reversibly-binding tethers.

## Results

**Figure 1:**
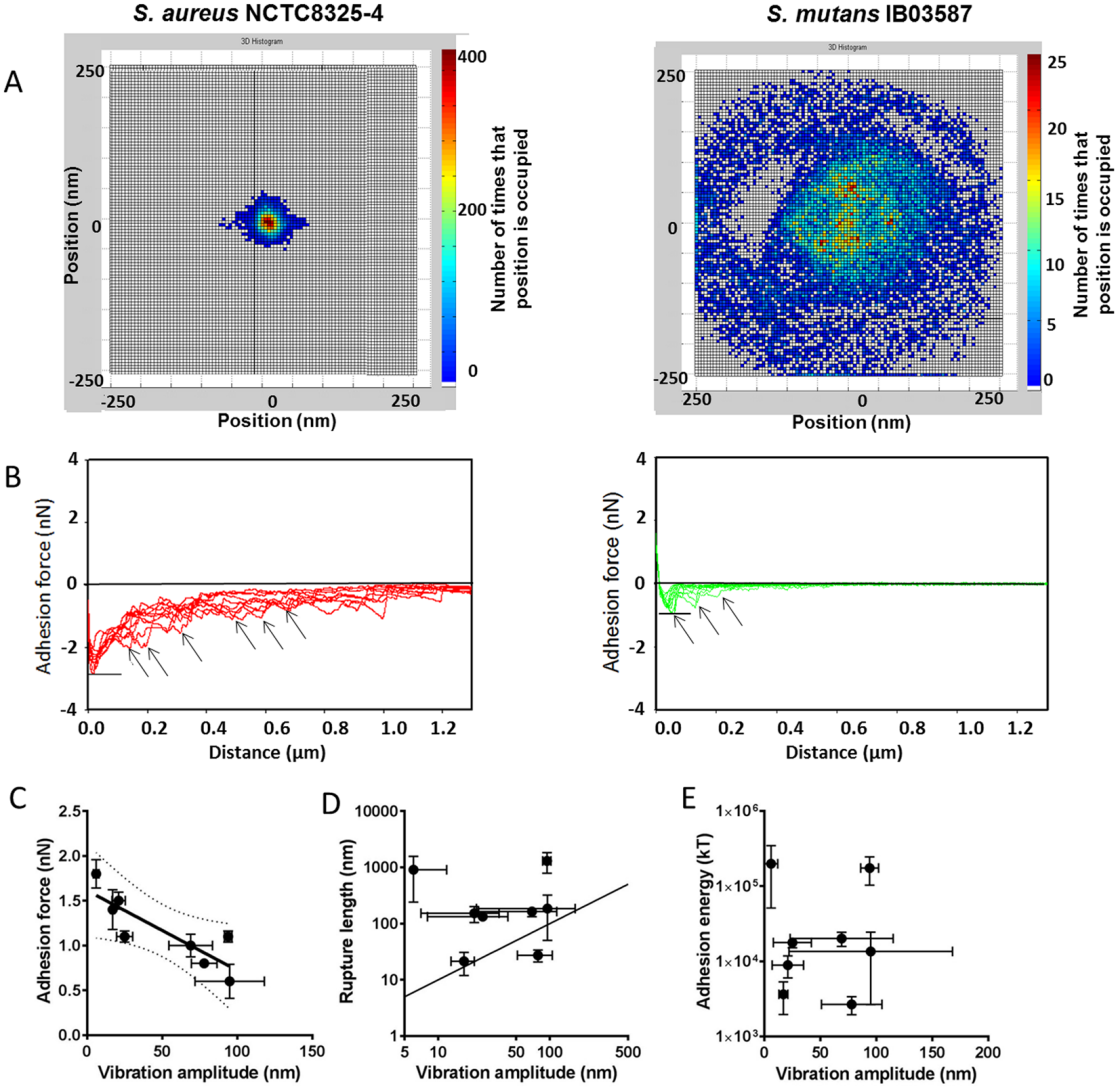
Bacterial adhesion forces, rupture lengths and energies for a variety of coccal strains and species as a function of their vibration amplitudes when adhering to a glass substratum. (**A**) Two examples of nanoscopic position-maps for *S. aureus* NCTC8325-4 and *S. mutans* IB03987 adhering to a glass surface, from which vibration amplitudes can be calculated. Pseudo-colors indicate the number of times a position was found occupied by the bacterium. (**B**) Two examples of force-distance curves as in single-bacterium contact probe AFM for *S. aureus* NCTC8325-4 and *S. mutans* IB03987 on a glass surface. The strongest force recorded during retraction, indicated by the horizontal line, is taken as the adhesion force^20^, while arrows indicate local minima indicative of detachment of different individual tethers (“adhesion peaks”^20^). Different lines of the same color indicate multiple measurements with one bacterial probe, taken in a random time sequence over the glass surface with respect to the lines. (**C**) Bacterial adhesion forces as a function of vibration amplitudes for different bacterial strains and species (see also Table 1). Error bars denote the standard errors of the means (see also Table 1). The drawn line indicates the best fit to a linear function with a correlation coefficient R^2^ of 0.72 (p < 0.01). Dotted lines indicate the 99% confidence limits. (**D**) Same as panel C, now for the rupture length as taken from the retract part in the force-distance curves. The line indicates the line of identity, where rupture lengths and vibration amplitudes match. (**E**) Same as panel C, now for the adhesion energy, i.e. the area under the force-distance curves.

Confined Brownian-motion of bacteria and abiotic particles adhering to surfaces becomes manifest as nanoscopic vibrations, forming a more or less circular pattern around an equilibrium position (Fig. 1A), the amplitude of which can be quantitated using Tethered Particle Motion analysis^12, 13^, called vibration spectroscopy in bacterial applications^15, 17, 18^. Bacterial probe AFM has become the method of choice to obtain bacterial adhesion forces with substratum surfaces^19^. From the two examples of force-distance curves presented in Fig. 1B, it can be seen from local minima (also called “adhesion peaks”^20^) that detachment of different individual tethers occurs when the bacterial AFM probe is removed further away from a glass surface. In the two examples given, multiple force-distance curves for each strain were taken with one bacterial probe in a random time sequence on a glass surface, varying by approximately 1 nN for the *S. aureus* NCTC8325-4 and less for *S. mutans* IB03987 strain. This is a similar or even smaller variation than observed for otherwise prepared single-bacterium contact AFM probes^20^. Also, our single-bacterium contact adhesion forces are comparable with those obtained using differently prepared single-bacterium contact AFM probes, although rigid comparisons cannot be made in microbiology as each laboratory has different culturing and harvesting procedures, while not only strains should match but also experimental conditions and substratum material. However, our adhesion forces for the two strains of *S. aureus* included (1.1 and 1.4 nN, see Table 1) on a hydrophilic glass surface compare reasonably well with those found for an *S. aureus* strain on a hydrophobic silicon wafer (8 nN)^21^, especially considering the difference in substratum hydrophobicity between the two studies. Data on *S. epidermidis* adhesion to hydrophilic glass (for our two *S. epidermidis* strains ranging between 0.8 and 1.5 nN) for another single-bacterium contact AFM probe preparation method and after 10 s bond-maturation^20^, are higher, but of the same order of magnitude as found here. For a *Lactobacillus plantarum* strain, a species not included in our study, to a self-assembled, hydrophobic monolayer adhesion forces widely ranged from 0.25 to 2.5 nN^22^. The broad correspondence between our single-bacterium contact adhesion forces and those found in the literature obtained with different single-bacterium contact AFM probe preparation methods, combined with the fact that our adhesion forces are never higher than reported in the literature, confirm that single-bacterium contact adhesion forces were measured with our probe preparation method employed (see also Supplementary Material, Fig. S1, presenting a new and simple method to demonstrate single-bacterium contact in AFM measurements with bacterial probes).

**Table 1:** Summary of results from analyses of confined Brownian-motion of adhering coccal bacteria and bacterial probe AFM for the different strains involved in this study^a^.

BACTERIAL STRAIN	BACTERIAL PROBE AFM	CONFINED BROWNIAN-MOTION ANALYSES		
	Adhesion force (nN)	Vibration amplitude (nm)	Autocorrelation time (s)	α^f^
*Staphylococcus aureus* ATCC12600	1.1 ± 0.1^b^	25 ± 5	0.3 ± 0.3	0.30 ± 0.02
*Staphylococcus aureus* NCTC8325-4	1.4 ± 0.2	17 ± 1^c^	0.1 ± 0.1	0.20 ± 0.01
*Staphylococcus epidermidis* ATCC35983	0.8 ± 0.1^d^	78 ± 9	0.2 ± 0.2	0.17 ± 0.01
*Staphylococcus epidermidis* ATCC35984	1.5 ± 0.1^c^	21 ± 4^c^	0.2 ± 0.2	0.23 ± 0.03
*Streptococcus mutans* LT11	1.8 ± 0.2	6 ± 2^e^	0.02 ± 0.01	0.07 ± 0.01
*Streptococcus mutans* IB03987	0.6 ± 0.2	95 ± 23	0.3 ± 0.1	0.18 ± 0.01
*Streptococcus salivarius* HB7	1.1 ± 0.1	94 ± 3	0.4 ± 0.5	0.17 ± 0.01
*Streptococcus salivarius* HBC-12	1.0 ± 0.1	69 ± 15	0.04 ± 0.02	0.10 ± 0.01

Autocorrelation times were taken as the time after which 50% of the autocorrelation was lost, while the α values presented refer to A × t^α^ (see also Eq. 4) for times t > 1 s. ± Signs indicate the standard errors of the mean over 10 different bacterial probes in AFM each used up to 5–10 times on different locations on a glass surface or 30–50 individual bacteria in bacterial vibration spectroscopy respectively, as divided over three separate bacterial cultures. ^a^Motile bacterial strains and species were excluded as self-induced movement, as e.g. by flagellated strains^39^ will interfere with their random Brownian-motion. ^b, c, d, e^These data points have been published before in^15, 17, 25, 40^ respectively. ^f^α = 1 indicates free diffusion with no confinement; α = 0 indicates confinement due to permanently attached tethers.

Further analysis of available literature data on nanoscopic bacterial vibration amplitudes and adhesion forces from eight different coccal bacterial strains, equally divided over four different species and complemented (where necessary) with new experimental data (Table 1), yielded a significant, linear (Pearson’s two-tailed correlation coefficient to R^2^ = 0.72, p < 0.01) increase of bacterial vibration amplitudes with decreasing adhesion forces of bacteria adhering on glass (Fig. 1C). The relation between adhesion forces acting perpendicular to a surface and vibration amplitudes of bacterial displacements parallel to a substratum surface, suggests involvement of detachment processes facilitating nanoscopic vibrations of adhering bacteria, while remaining microscopically adhering at the same position. Rupture lengths in AFM force-distance curves as arising from the forced contact and separation of adhering bacteria from their substratum surfaces, were always similar or up to 100x larger than vibration amplitudes induced by the relatively small, Brownian-motion forces (Fig. 1D). No relation existed between rupture lengths and vibration amplitudes, because not all bacterial strains have tethers with the same length and same spring constant. Moreover, the forced contact and separation in AFM may have irreversibly affected the length and elasticity of the tethers. Similarly as rupture lengths, adhesion energies bear no relation with bacterial vibration amplitudes on glass (Fig. 1E). In Fig. 1E it can also be seen, that adhesion energies of entire bacteria are much larger than 1.5 kT, confirming their irreversible adhesion as a whole, while the energies by which individual tethers adhere are hard to reliably estimate but are clearly much smaller (see Fig. 1B). However, even dividing the adhesion energies obtained over 100 tethers, representing an over-estimation by comparison with Fig. 1B, would yield adhesion energies of tethers that are too large to allow their individual detachment. However, several papers have demonstrated, that compared with other approaches, AFM overestimates adhesion forces and energies by a factor of 10^2^ to 10^5^ ^11^. Bacterial detachment experiments under fluid flow for instance, have indicated that forces to detach bacteria from a glass surface vary between 0.3 to 5.4 pN for *S. epidermidis* strains^11^. DLVO calculations^23^ yielded attractive forces towards the secondary minimum between 0.05 to 0.1 pN for *Raoultella terrigena* and *S. epidermidis* strains adhering to glass, with adhesion energies up to 1 kT. Single tether adhesion forces were measured using optical tweezers and found to be around 20 pN for an *S. aureus* strain with fibronectin-binding proteins to fibronectin-coated beads^24^. Likely, the forced contact under an applied loading force during AFM measurements creates adhesion forces in excess of the forces occurring under more natural conditions. Even AFM adhesion force measurements as a function of loading force and their subsequent linear extrapolation to zero loading force, did not yield adhesion force values comparable with the above ones^25^. Conservatively assuming that AFM overestimates bacterial adhesion forces and energies by a factor of 10^3^, this yields adhesion energies of 2 to 300 kT, which is still high for bacteria to detach as a whole under influence of thermal energy. However, applying the same factor to the bacterial adhesion forces measured (Fig. 1B), yields adhesion forces in the pN range, comparable with the force values obtained using other methods^11^. Assuming 100 binding tethers to be involved in the adhesion of a single bacterium, this yields tether adhesion forces in the order of 10^−2^ pN and tether adhesion energies that allow individual tethers to detach by thermal energy only.

**Figure 2:**
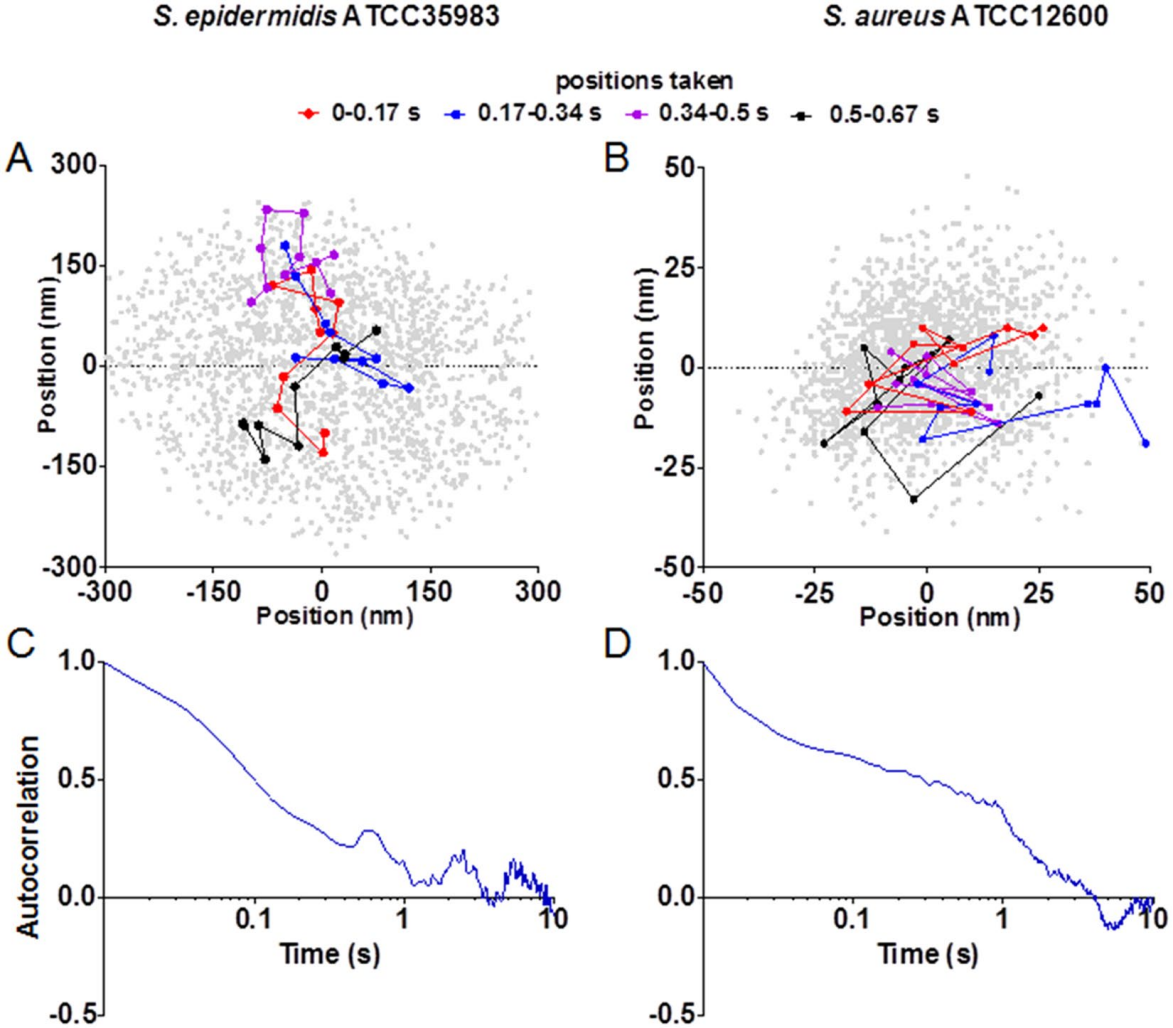
Time-resolved nanoscopic position-maps and autocorrelation functions of *S. epidermidis* ATCC35983 and *S. aureus* ATCC12600 adhering to a glass surface. (**A**) Time-resolved position map of *S. epidermidis* ATCC35983 adhering to a glass surface over a 33 s measuring period (bacterial positions indicated by grey dots), resolved into four time intervals of 0.17 s (equivalent to ten frames) with bacterial trajectories and positions indicated by colored lines and dots for the different intervals. (**B**) Same as panel A, now for *S. aureus* ATCC12600. (**C**) Autocorrelation as a function of time calculated over the entire 33 s measuring period from the x-positions of *S. epidermidis* ATCC35983 adhering to a glass surface (displayed only for the first 10 s of an experiment). (**D**) Same as panel (**C**), now for *S. aureus* ATCC12600.

Time-resolved analyses of bacterial position-maps indicated that vibrating bacteria do not move randomly from one side of the position-map to another, but reside for short periods of time in the same region of the position-map (Fig. 2A and B). Autocorrelation functions of bacterial positions according to Eq. 2 showed that bacterial positions are nanoscopically correlated (Fig. 2C and D). Autocorrelation reduced over time and got fully lost only after 5 to 10 s, while for the collection of strains used in this study 50% of the autocorrelation remained to exist for several tenths of seconds (see also Table 1). This attests to the dynamic detachment and successive re-attachment of the tethers to their initial positions for prolonged periods of time, therewith not only adhering but also immobilizing bacteria to the substratum surface.

**Figure 3:**
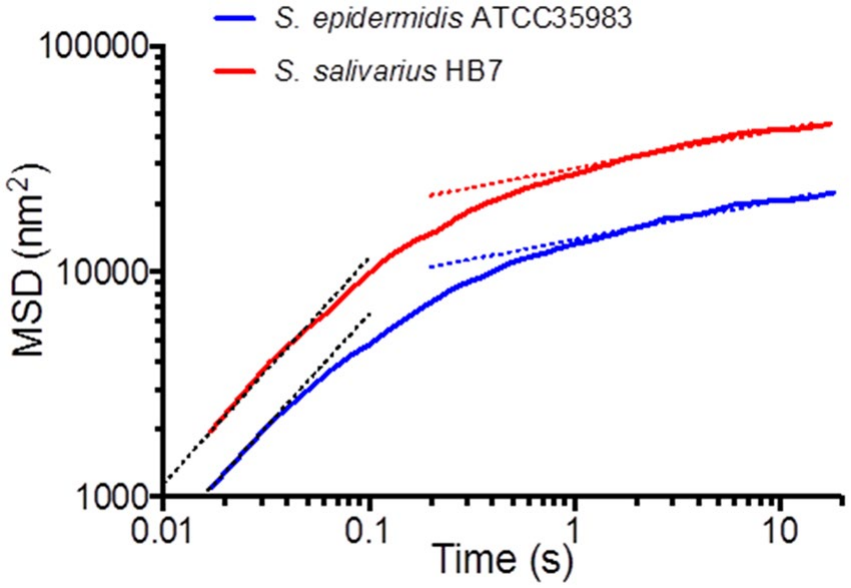
Mean Squared Displacement (MSD) as a function of time for two different bacterial strains involved in this study. Black dotted lines represent MSD(t) = A × t^α^ (see also Eq. 4) corresponding with α-values equal to unity, while colored dotted lines are fitted to measured MSD values for t > 5 s (for α-values see also Table 1).

Vibrating bacteria adhering on a substratum surface have indeed seldom been observed to displace themselves microscopically to another position on a substratum surface, unless vibration amplitudes amounted 250–300 nm in which case an entire bacterium can be observed over time to displace itself several micrometers over the surface to a new position, but never detach itself from the surface. Vibration amplitudes of 250–300 nm are rare however, and have only been observed for few individual bacteria within one experiment but never over the majority of bacteria adhering and vibrating in one experiment. Most adhering bacteria vibrated with strain-specific amplitudes less than 250 nm (Table 1) and their possible displacements are extremely hard to discern microscopically by the unaided eye without the use of image analysis software. However, within the framework of our hypothesis that bacteria adhere irreversibly to surfaces through multiple, reversibly-binding tethers that detach and successively re-attach, demonstration of nanoscopic displacements over time of adhering bacteria vibrating with smaller amplitudes would provide additional evidence of dynamic tether detachment and re-attachment. One way to determine possible displacements in a more sensitive way is to determine the Mean Squared Displacement (MSD) of adhering bacteria (see Eq. 3). Figure 3 shows two examples of the MSD as a function of time for two bacterial strains involved in this study. MSD values for both strains shown, leveled off within 10 s, but importantly their squared displacement continued to increase slowly over time. The MSD of colloidal particles in viscoelastic media increases proportionally with time to the power α (see Eq. 4), with an α value of unity corresponding with diffusion-controlled motion in a viscous fluid. Figure 3 shows that the confined Brownian-motion of adhering bacteria was initially purely diffusive with α equaling unity, but α gradually reduced over time but never reached 0, regardless of the strain involved (see also Table 1).

## Discussion

In this paper we aim to demonstrate that bacteria adhere to substratum surfaces through multiple, reversibly-binding tethers that detach and successively re-attach, but never detach all at the same time, resulting in irreversible bacterial adhesion. In order to provide general validity to our conclusions, the study was carried out with four different bacterial species, each represented by two strains of varying origin and highly diverse, known surface characteristics (see Table 2). Although all eight strains included in this study were chosen to be cocci because they show simpler vibration patterns, we believe this conclusion to have a more general validity, extending to rod-shaped organisms as well. Earlier we demonstrated that there were no systematic differences between vibration amplitudes of Gram-positive and Gram-negative strains^15^. Clearly, our conclusion does not hold for motile bacterial strains and species because of their self-induced movement.

**Table 2:** Overview of the origin and relevant, known surface characteristics of the eight strains used in this study, arranged with each of the four species grouped together.

BACTERIAL STRAIN	ORIGIN	SURFACE CHARACTERISTICS			REFERENCE
		Outermost surface structures	Zeta potential (mV)	Water contact angle (degrees)	
*Staphylococcus aureus* ATCC12600	Pleural fluid	EPS layer	−25	30	41, 42
*Staphylococcus aureus* NCTC8325-4	Isogenic mutant *S. aureus* NCTC8325	Fibronectin binding protein	—	—	43, 44
*Staphylococcus epidermidis* ATCC35983	Human blood	EPS layer	−51	38	11, 45, 46
*Staphylococcus epidermidis* ATCC35984	Catheter sepsis	EPS layer	−30	40	42, 45, 46
*Streptococcus mutans* LT11	Variant from *S. mutans* UA159	Antigen I/II	−13	29	47, 48
*Streptococcus mutans* IB03987	Isogenic mutant *S. mutans* LT11	No antigen I/II	−28	33	48
*Streptococcus salivarius* HB7	Isogenic mutant *S. salivarius* HB	Fibrillated, fibril length 91 nm	−18	30	49
*Streptococcus salivarius* HBC-12	Isogenic mutant *S. salivarius* HB	no fibrils	−19	21	49, 50

Single-bacterium contact probe AFM (Fig. 1B) confirmed that a bacterium adheres through different individual tethers, which is as such not new. Thewes *et al*.^21^ proposed a model in which adhering bacteria were tethered to a surface through multiple springs by forcing contact between a strain of *S. aureus* and a surface using an AFM cantilever, and equally so forcing detachment of tethers by retracting the cantilever yielding multiple adhesion peaks in the retract force-distance curve and a single rupture of the entire bond. Essentially different from the study of Thewes *et al*.^21^, we studied vibrations and displacements of adhering bacteria occurring through successive detachment and re-attachment of multiple tethers occurring under the naturally prevailing adhesion and Brownian-motion forces. This is different than the forced adhesion and detachment in AFM studies^21^, not allowing any subsequent re-attachment of a once detached tether, as is possible in bacterial vibration spectroscopy. The relation between perpendicularly acting adhesion forces on the individual tethers and vibration amplitudes confirms that detachment of individual tethers occurs while bacteria adhere to a substratum surface (Fig. 1C), but apparently never all at the same time. Elsewise an entire bacterium would detach. Therewith bacterial adhesion becomes essentially irreversible, despite reversibly-binding tethers. The relation between bacterial adhesion forces and vibration amplitudes may be interpreted as reflecting the frequency with which tethers detach and re-attach. This interpretation is inspired by the simulation program presented, in which the adhesion force of an individual binding tether presents a threshold for detachment. This threshold force only allows detachment of the tether once stepwise increases of the elastic force arising from the tether due to its elongation, exceeds the threshold. Viewed from this perspective, smaller adhesion forces reflect easier, more frequent detachment and sucessive re-attachment, yielding larger displacements and vibration amplitudes.

The maintenance of nanoscopic positon by adhering bacteria for several tenths of seconds indicated by their 50% loss of autocorrelation times (Table 1) and by full loss of autocorrelation only after several seconds (Fig. 2C and D), confirms that tethers will re-attach to the same preferential sites on a substratum surface, at least for a certain period of time. Incidentally, the existence of preferential re-attachment sites has been demonstrated on a more macroscopic basis by repeatedly allowing inert, micrometer-sized polystyrene particles to adhere to a glass surface followed by detachment by passing liquid-air interfaces^26^. In each and every new round of an experiment, image analysis indicated that exactly the same, initially occupied adhesion sites were first occupied again^27^, proving existence of preferential adhesion sites, even on a glass surface. Yet, analysis of MSD values of adhering bacteria indicate slow, nanoscopic displacements over time, similar as exhibited by silica particles in transient polyethylene polymer networks, for which it was concluded that silica particles attach to network molecules with non-permanent cross-links^28^. This is in line with the detachment and successive re-attachment of individual bacterial tethers, as the likelihood of a detached tether finding back the same preferential nansocopic site for re-attachment decreases over time, especially when vibrations occur over longer distances.

**Figure 4:**
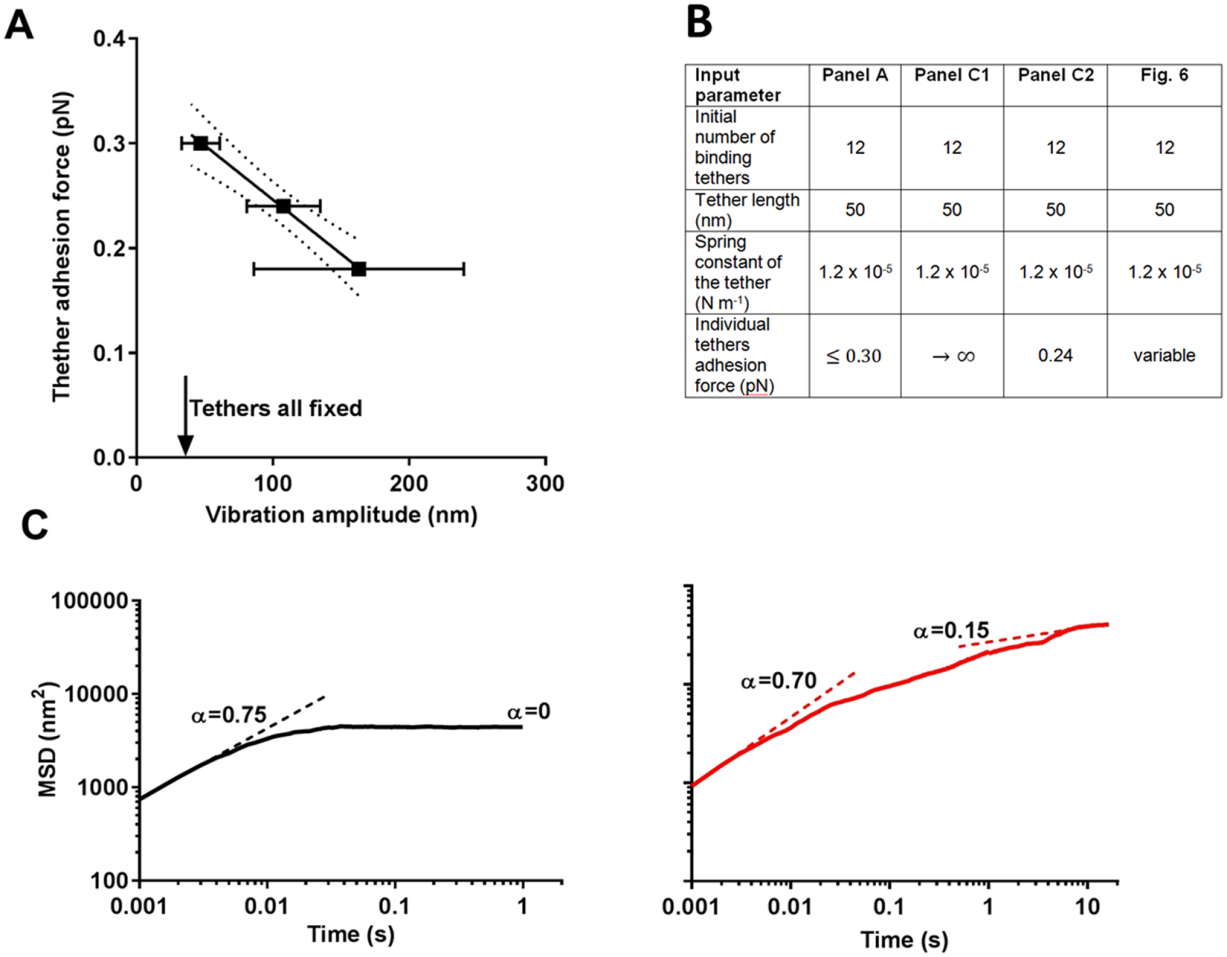
*In silico* modeling of tethered particle motion, accounting for detachment and successive re-attachment and attachment of additional tethers. (**A**) Particle adhesion forces as a function of vibration amplitudes derived from simulated position-maps, according to the *in silico* input parameters listed in panel B. The drawn line indicates the best fit to a linear function with a correlation coefficient R^2^ of 1.0 (p < 0.05). Dotted lines indicate the 95% confidence limits. Error bars in vibration amplitudes indicate standard deviation over 7 simulated particles monitored over a 33 s time interval, while *in silico* errors in adhesion forces are absent. When simulations are run in which the tether adhesion force is set to approach “infinity”, therewith inhibiting detachment, a simulated vibration amplitude of 33 nm results. (**B**) Summary of particle-related input parameters for the simulated results in panels A and C and in Fig. 6. (**C**) Mean Squared Displacement (MSD) obtained from simulated position-maps as a function of time for adhering particles initially attaching with 12 binding tethers without (left panel, C1) or with (right panel, C2) the possibility of tethers to detach and re-attach or to invoke new tethers in particle adhesion. Simulated MSDs as a function of time were fitted to MSD(t) = A × t^α^ (see also Eq. 4), yielding initial α-values close to unity for tethers without the possibility to detach, indicative of diffusion-controlled particle motion. For t > 5 s, simulated α-values are zero for tethers programmed to be unable to detach, while for dynamically binding tethers 0 < α ≪ 1 (for experimental α-values see Table 1). Simulated MSD values presented each involve 6 particles.

Existing *in silico* modeling of tethered particle motion trajectories by others neglected the possible detachment and subsequent re-attachment of tethers^29^. Therefore we wrote a new, *in silico* model of tethered particle motion, including the possibility of detachment and successive re-attachment of individual tethers (for details see Supplementary Material: *In silico* modeling of tethered particle motion with detaching and successively re-attaching tethers), extending an earlier Brownian-motion tethered particle motion simulation model^29, 30^. The extended model was used to confirm the key-observations underlying the experimental evidence provided in support of our hypothesis. Figure 4A summarizes vibration amplitudes obtained from simulated position-maps of adhering particles *versus* their adhesion force for particles adhering to a surface with initially 12 binding tethers, each possessing a spring constant of 1.2 × 10^−5^ N m^−1^. This value represents an average over different bacterial strains, as previously obtained using bacterial vibration spectroscopy^15^ (see Fig. 4B for a summary of particle-related input parameters to the simulation). The relation between tether adhesion forces and simulated particle vibration amplitudes (Fig. 4A) fully confirms the experimentally observed relation in Fig. 1C. Interestingly, the analysis of simulated tethered particle motion yields similarly large standard deviations in vibration amplitudes as obtained experimentally. This attests to the highly stochastic nature of the process. Next, the model was used to confirm that ongoing, slow nanoscopic displacement of adhering particles after prolonged periods of time (Fig. 3) can only be explained if detached tethers are allowed to re-attach or tethers not involved in initial particle adhesion, become within reach of the substratum surface and attach. In the modeling results presented in Fig. 4C, it can be clearly seen that tethered particle motion in absence of detachment and (re-)attachment possibilities does not yield the continued, slow increase in MSD values after prolonged periods of time, that we observed experimentally (compare Figs 3 and 4C). The validity of our simulation model can be inferred first of all from the exponent *α* describing whether initial displacement is diffusion-controlled: calculation of the exponent *α* for the initial displacement of simulated particle motion yielded values close to unity (Fig. 4C), thus indicating that our simulations indeed yield diffusion-controlled initial displacement of tethered particles, fully in line with our experimental results (Fig. 3). A second validation follows from the α value after prolonged periods of time, amounting around 0.15 for simulated tethered particle motion (see also Fig. 4C), which compares well with the values obtained experimentally for the collection of bacterial strains used (compare Fig. 4C and Table 1) and indicative of ongoing confined displacement.

**Figure 5:**
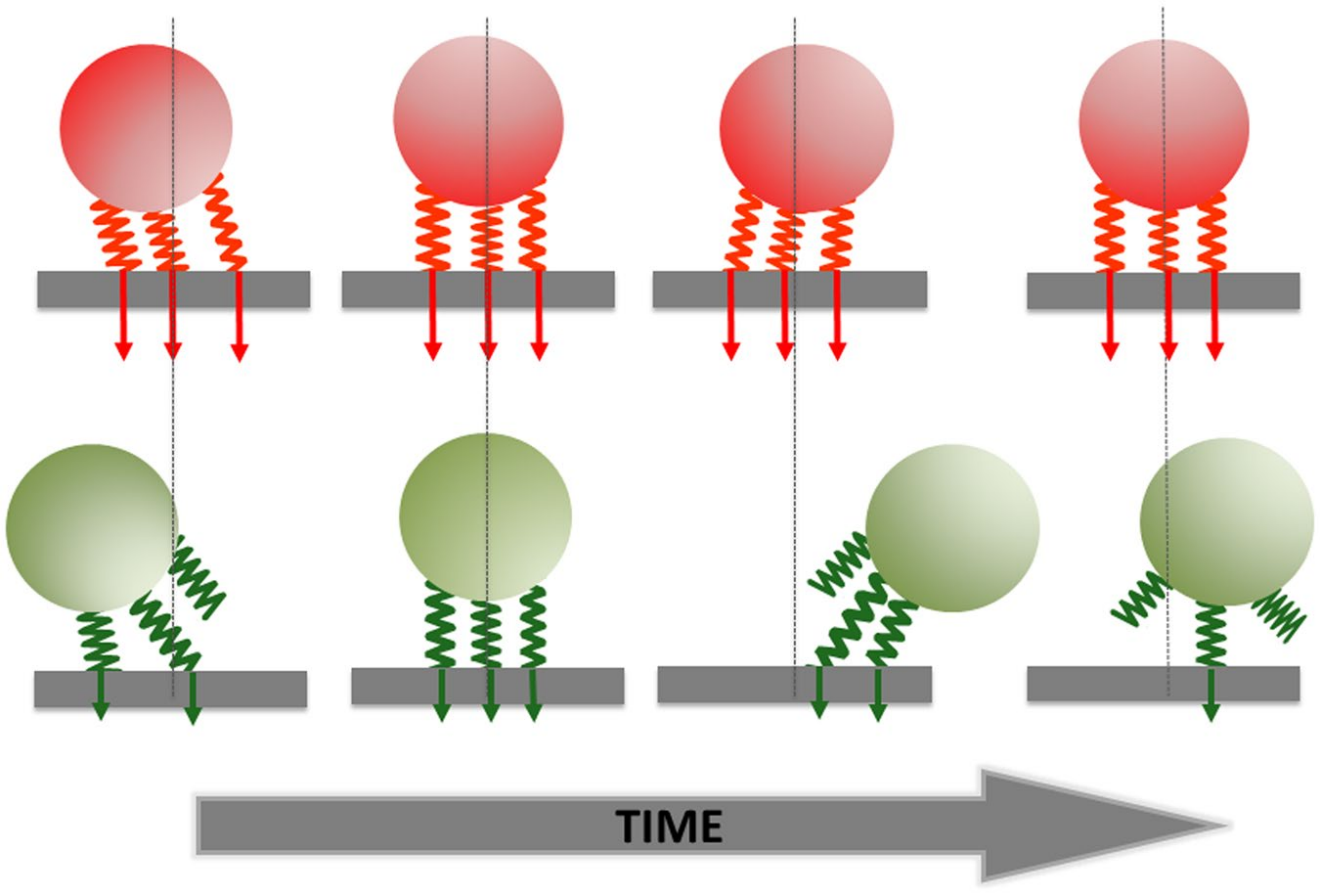
Proposed mechanism of the irreversibility of bacterial adhesion due to multiple, reversibly binding tethers as derived from the influence of adhesion forces on the vibration amplitude and confined Brownian-motion of adhering bacteria and supported by *in silico* simulations: Strong adhesion forces (red panel) of individual tethers impede Brownian-motion induced detachment of tethers resulting in small vibration amplitudes, while small adhesion forces (green panel) allow detachment of tethers resulting in larger vibration amplitudes and nanoscopic displacement parallel to the surface when re-attaching at a different position.

Collectively, these experimental observations and *in silico* modeling confirm our hypothesis that bacteria adhere to surfaces through multiple, reversibly-binding tethers that detach and successively re-attach but never all at the same time and not always to the same nanoscopic position, depending on their vibration amplitude (Fig. 5). Said differently, we attribute the irreversibility of bacterial adhesion to reversibly binding tethers and subsequently explain how this can cause nanoscopic displacement of an adhering bacterium. This relates to a previously introduced concept of mobile and immobile adhesion of bacteria to surfaces^31^. The expression “mobile adhesion” was proposed for bacteria that exhibited macroscopic displacement, microscopically visible by the unaided eye without the use of image analysis software, prior to becoming immobilized on a fixed adhesion site. Immobile adhesion” was defined as bacteria adhering without showing any such macroscopic displacement. Interestingly, mobile adhesion occurred much more frequently on a hydrophilic than on a hydrophobic surface. Although the displacement referred to in mobile adhesion extended over the micrometer-range, it is speculated here that during the initial, mobile adhesion phase, the number of binding tethers increases to its final number. This speculation is confirmed by *in silico* modeling of the increase in the number of binding tethers from its initial number to a final number at the end of the experimental timeframe (see Video V1 in Supplementary Material). Since bacterial adhesion forces on hydrophobic surfaces are generally larger than on hydrophilic ones, a comparison of the changes over time of bacterial vibration amplitudes on hydrophobic and hydrophilic surfaces might confirm this speculation, although likely not all bacteria in a population will adhere with exactly the same adhesion force and therewith not the same detachment frequency and number of binding tethers. This cannot only be concluded from the number of local minima in AFM force-distance curves upon retract (see Fig. 1B) that can differ for different individual bacteria of the same strain (data not shown), but also from occasionally occurring position-maps of adhering bacteria that are aberrant from circularly occurring position-maps.

**Figure 6:**
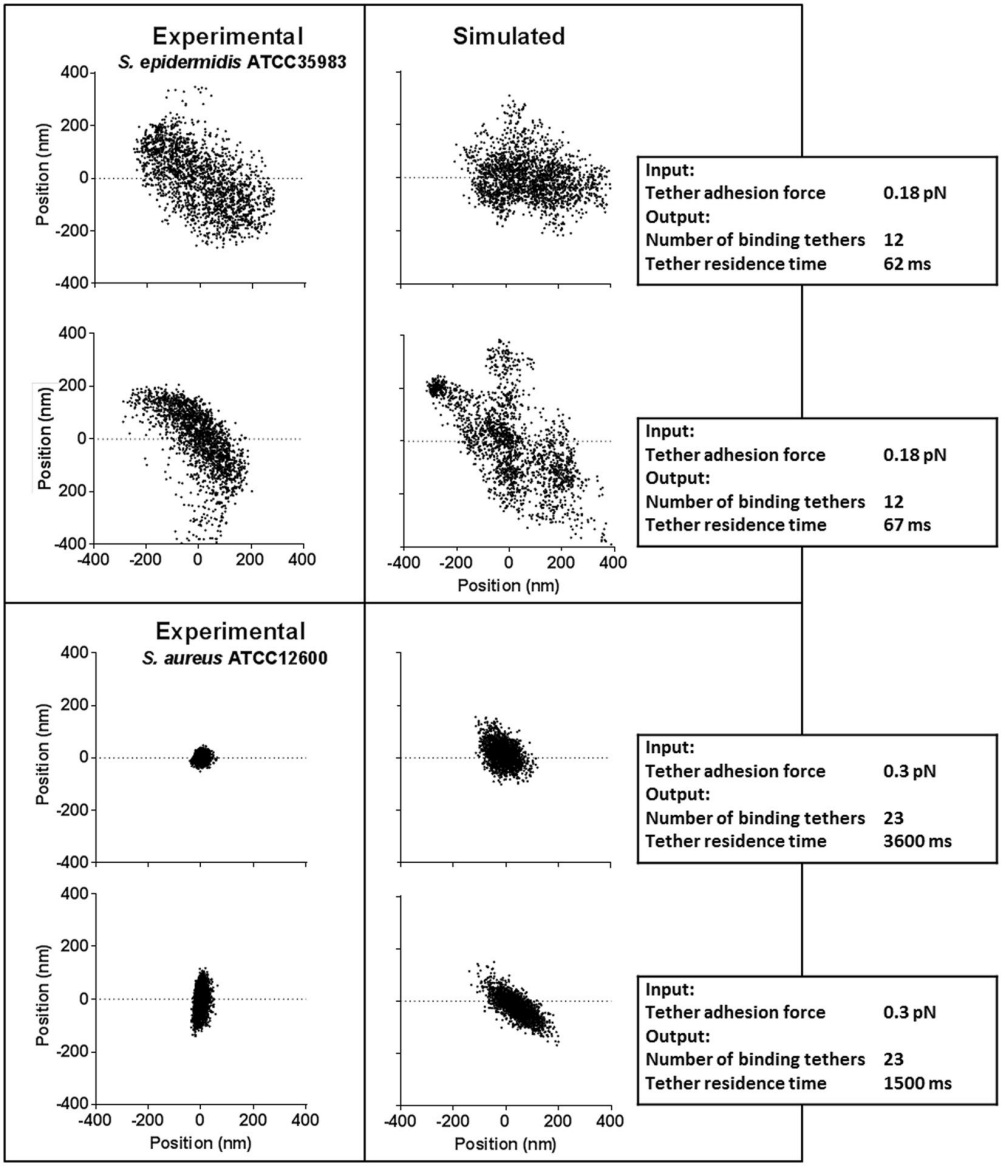
Experimentally observed position-maps of individual bacteria of different strains (left panels), as compared with *in silico* (right panels) generated position-maps obtained by varying the tether adhesion force and therewith the resulting number of binding tethers per bacterium. Input parameters are listed in Fig. 4B, while the tether adhesion force used in the simulation and the resulting final number of binding tethers are given in the simulated position-maps. For each strain, a circular position-map and an aberrant one is presented.

In Fig. 6, we have compiled a number of such experimentally observed, aberrant position-maps, not representative for the majority population of a given strain and varied the tether adhesion force to yield matching, simulated position-maps. *In silico* modeling confirms that both the circular position-maps of the majority of the population as well as aberrant maps can be modelled by varying the tether adhesion force, impacting the tether residence time and their final number. Thus not all bacteria within a population will bind with the same tether adhesion force and final number of binding tethers.

Considering irreversible bacterial adhesion as resulting from a succession of detachment and re-attachment of reversibly-binding tethers, such as provided by bacterial cell surface appendages or extruding EPS, the discussion on the reversibility or irreversibility of bacterial adhesion is herewith put a par with the (ir)reversibility of adsorbed proteins. Proteins are known to adsorb through multiple molecular segments that each individually adsorb in a reversible fashion^32^, with estimated residence times between milli- and nano-seconds, depending on the affinity of binding between the segment and the substratum (private communication M.A. Cohen-Stuart and W. Norde). Especially higher molecular weight proteins, possessing numerous adsorbing segments, adsorb more irreversibly than smaller ones due to the higher statistical unlikeliness that all molecular segments will simultaneously detach in a high-molecular weight protein, a phenomenon known as “the Vroman effect”^33^. Residence times of individual bacterial tethers on a substratum surface are currently out of experimental reach, but the times after which autocorrelation of bacterial positions reduce to 50% (Table 1) suggests that residence times of the individual tethers may be in the order of milli-seconds, which is possibly higher than estimated for molecular protein segments. However, the bacterial tethers on their turn will attach with multiple molecular segments, which explains why residence times of bacteria would be in the second range. Simulations presented in Fig. 6 confirm that indeed tether residence times can vary from milli-seconds to seconds, strongly dependent on the tether adhesion force.

Summarizing, it is demonstrated that bacterial adhesion involves multiple reversibly-binding tethers that detach from and successively re-attach to a surface, resulting in irreversible adhesion of an organism as a whole. The analyses of confined Brownian-motion of adhering bacteria, AFM force-distance curves and *in silico* modeling played a pivotal role in providing evidence in support of the hypothesis leading to this conclusion. Revealing the exact number and adhesiveness of reversibly-binding tethers and dynamics of their individual Brownian-motion induced detachment-(re-)attachment constitutes a new challenge in the physico-chemistry of bacterial adhesion to surfaces, that might offer new possibilities for engineering anti-fouling surfaces to which end bacterial vibration spectroscopy, although in its infancy, will be an important tool. As an on-set to this, it was shown that antimicrobial treatment of adhering, oral bacteria yielded smaller vibration amplitudes coinciding with reduced detachment of adhering bacteria by a passing liquid-air interface, from which it was concluded that Brownian-motion assisted detachment of adhering bacteria from a surface^18^.

## Materials and Methods

### Bacterial growth and harvesting

Eight coccal bacterial strains, representing four species, were chosen for this study and grown on blood agar from frozen stock (−80 °C). Details on the origin and known, relevant surface characteristics of the strains are listed in Table 2.

Single colonies were inoculated into 10 mL of TSB (Tryptone Soya Broth, OXOID, Basingstoke, UK) for staphylococci or THB (Todd Hewitt Broth, OXOID) for streptococci and incubated for 24 h prior to transfer into 200 mL main cultures. After 16 h, bacteria were harvested by centrifugation (3 × 5000 *g*) and either washed and suspended in a low ionic strength buffer (0.5 mM potassium chloride, 0.02 mM potassium phosphate, 0.01 mM calcium chloride, pH 6.8) when prepared for bacterial vibrations experiments, or washed and suspended in 10 mM potassium phosphate buffer, pH 6.8 for bacterial probe AFM. Bacterial suspensions were sonicated in an ice/water bath for 3 × 10 s (Vibra Cell model 375; Sonics and Materials, Danburry, CT, USA) with 30 s breaks in between to break up aggregates. Bacterial vibrational spectroscopy and AFM were both carried out in ionic strength suspensions that optimally enhanced differences between the different strains and species in order to obtain more meaningful relations. For bacterial vibration spectroscopy, bacteria were suspended to a concentration of 3 × 10^8^ bacteria mL^−1^, determined using a Bürker-Türk counting chamber, while for the preparation of single-bacterium contact AFM probes, a 100-fold lower concentration was used. Physiological status (live-dead status, i.e. absence of membrane-damage) was similar for all strains and species during both AFM experiments and vibration spectroscopy, while metabolic activity was slightly less during vibration spectroscopy but only so for the *S. mutans* strains (see Table S1).

### Glass substrata

All experiments were carried out on glass surfaces. Glass surfaces were commercially purchased (Menzel GmbH, Braunschweig, Germany) as microscope slides with dimensions 76 × 26 × 1 mm. Prior to all experiments, glass surfaces were cleaned in 2% RBS solution (Omniclean RBS 35, Breda, The Netherlands) in an ultrasonic bath and rinsed thoroughly with tap water, methanol and ultrapure water, yielding a water contact angle of less than 10 degrees. X-ray Photoelectron Spectroscopy yielded an elemental surface composition with respect to oxygen and silicon of 56 ± 1 and 27 ± 1 at%, matching the ratio as expected on basis of the molecular composition of glass and a minor carbon contamination of 8 ± 1 at%. All glass slides were used in experiments immediately after cleaning.

### Bacterial probe AFM adhesion force measurements

Bacterial adhesion forces were measured on glass surfaces following a generally applied procedure^25^ at a an approach/retract velocity of 2 µm s^−1^, albeit with a slight modification to ensure single-bacterium contact during AFM force measurements^20, 34^. Briefly, cantilevers were calibrated for their stiffness using AFM Tune cIT v2.5 software. The deflection sensitivity, *α*_*s*_, of the cantilever was recorded on bare glass to calculate the applied normal force, *F*_*n*_, using

1 $${F}_{{\rm{n}}}={\alpha }_{s}x{V}_{n}x{K}_{n}$$

where *K*_*n*_ is the cantilever stiffness (0.046 ± 0.002 N m^−1^; average over 26 cantilevers) and *V*_*n*_ is the voltage output from the AFM photodiode due to cantilever deflection. Bacteria were immobilized on the tipless cantilevers (NPO, Bruker AFM Probes, Camarillo, CA, USA) via electrostatic interaction with poly-L-lysine (PLL; Sigma-Aldrich, USA) using a micromanipulator (Narishige Groups, Tokyo, Japan)^35^. The far end of the cantilever was dipped in a droplet of PLL for 1 min and dried in air (2 min), followed by 1 min immersion in a droplet of greatly diluted, bacterial suspension (3 × 10^6^ bacteria mL^−1^) to let a bacterium adhere. In other studies, we used higher bacterial concentrations (3 × 10^8^ bacteria mL^−1^) that resulted in a lawn of bacteria on the cantilever. Nevertheless, such lawns never gave rise to double-contour lines in images taken with such probes (see Fig. S1A and B) and adequately imaged the dimensions of an AFM calibration grid (HS-20MG BudgetSensors, Innovative Solutions Bulgaria Ltd., Sofia, Bulgaria), albeit with a little distortion (Fig. S1B). Moreover, we seldom saw bi- or tri-modal distributions of adhesion forces measured. From this we concluded that the cell surfaces of different individual bacteria on a cantilever were never equidistant from the substratum surface within the range of their adhesion force and hence data could be interpreted as single-bacterium contact adhesion forces. In line with current trends in single-bacterium probe AFM^20, 22, 36^, we decided to use more diluted suspensions for bacterial probe preparation, which is more time-consuming but in more than 85% of all cases (in our experience) yields a single bacterium or sometimes very few bacteria present on the cantilever and single-contour lines in imaging without any distortion (Fig. S1C). Moreover, such single-bacterium contact AFM probes reflects the width of an AFM calibration grid equally well as an AFM tip (3.96 versus 3.95 µm, respectively), despite the fact that the AFM tip has a half-width of 5 nm at level 17.5 nm above the tip end, while the bacterial half-width at that level equals 122 nm (note that the bacterial probe for the multiple-bacteria probe yielded the smallest width (3.54 µm) for the AFM calibration grid). The use of a low bacterial concentration in suspension for bacterial probe preparation generally leads to the presence of a single or very few bacteria on a cantilever compared with the use of a high concentration bacterial suspension (see Fig. S1C). Incidentally, from Fig. S1C it can be seen that the majority of all bacteria thus attached to an AFM cantilever, including the bacterium on both single-bacterium probes, are green-fluorescent after LIVE/DEAD staining, indicative of the organism(s) being alive, or technically more correct: “not cell-wall damaged”^37^. Thus prepared single-bacterium contact AFM probes never exhibited multi-modal distributions of adhesion forces, while force-distance curves were highly different than obtained with a PLL-coated cantilever without attached bacteria (see Fig. S1D). Our force data can thus be interpreted as single-bacterium contact forces. Bacterial probes were used for AFM immediately after preparation in a wetted state. AFM measurements were conducted under a loading force of 5 nN, a velocity of 3 µm s^−1^ and at a “zero seconds” surface delay time (i.e. < 100 ms). The strongest force recorded during retraction of the cantilever is taken as the adhesion force.

### Bacterial vibration spectroscopy

Bacteria were allowed to adhere to the glass surface in a parallel plate flow chamber (75 × 17 × 0.75 mm) by circulating bacterial suspension through the chamber at a shear rate of 10 s^−1^ for 1 h, after which the system was rinsed with buffer to remove non-adhering bacteria and flow was arrested. Subsequently, 15 min after arresting the flow, videos of adhering bacteria were recorded with a CCD camera (A101F, Basler AG, Ahrensburg, Germany) mounted on a phase-contrast microscope (BH2-RFCA, Olympus Optical Co., Tokyo, Japan). The camera was coupled to an image analysis program (Matlab, The MathWorks, Natick, MA, USA). Each image with an 8 bit grey scale (256 grey-values), was reduced to 1392 × 128 pixels resulting in a frame rate of 60 consecutive images per second. Fourier transform analysis of bacterial displacements revealed that high amplitudes of Fourier components were observed only at low frequencies up to 5 Hz, which indicates that the effective frame rate of 60 Hz provides ample time resolution to capture bacterial vibrations^15^.

The centroid of a filter-enhanced image of the adhering bacterium, obtained by using Matlab software, was taken as its position. Positions were calculated using 2000 images per recorded bacterium, over a 33 s time interval (see also Fig. 1A) and plotted in a distribution histogram. The standard deviation of the position of each bacterium in the distribution histogram was taken as the vibration amplitude, assuming a circular position-map. Each experiment involved the analysis of the vibration of 30 randomly selected bacteria. In order to eliminate the influence of building vibrations, the vibrational amplitude of a fixed marker was calculated and subtracted from the vibrational amplitudes calculated for each bacterium yielding a spatial resolution of around 5 nm^15, 29^.

The autocorrelation of the nanoscopic positions of adhering bacteria in different time-resolved position-maps over the measuring period of 33 s (corresponding with 2000 frames) was obtained using Matlab software and indicates the correspondence of the bacterial positions as a function of the time lag between them^15^. Briefly, the autocorrelation *R(h)* between bacterial positions for different time lags was calculated according to

2 $$R(h)=\frac{{\sum }_{n=0}^{N-h-1}x(n)\,x\,x(n+h)}{{\sum }_{n=0}^{N-1}x(n)\,x\,x(n)}$$

where *x* is the bacterial *x*-position as related to the average *x*-position, *N* is the number of frames (2000) in a time series of 33 s and *h* is the time lag in steps (*h* consecutive frames) of 0.017 s. If the autocorrelation *R(h)* equals unity, the *x-*position at each time is identical to the position *h* time steps later.

The MSD as a function of time is indicative of the positional freedom bacteria experience^14^ and was calculated at different time points according to

3 $$\,MSD=\langle \langle \Delta {r}^{2}\rangle \rangle =\frac{1}{10}\sum _{k=1}^{M}\frac{1}{N}\sum _{i=1}^{N}{({r}_{i}-{r}_{j})}^{2}$$

where *N* is the number of frames in a time series, *r*_*i*_ the radial position of the bacterium in frame *i* and *r*_*j*_ the radial starting position at frame *j* with *i* > *j*. MSD values were calculated for *N* = 1000, corresponding to a time period of 16 s and averaged over series with *M* different radial starting positions (*M* = 10), 100 frames apart from each other. Finally, MSD values from 10 individual bacteria were averaged.

MSD as a function of time *t* can be described as

4 $${\rm{MSD}}({\rm{t}})={\rm{A}}\times {{\rm{t}}}^{{\boldsymbol{\alpha }}}$$

where *A* is a proportionality constant, *t* is time and the exponent *α* describes whether displacement is purely diffusion-controlled (α = 1) or confined by tethers attaching to a surface (α < 1) and approaching 0 after a certain period of time indicative of absence of further displacement^28^.

### *In silico* modeling of tethered particle motion with detaching and successively re-attaching tethers

In order to provide further evidence for our hypothesis that bacterial adhesion to surfaces becomes irreversible through multiple, reversibly-binding tethers that detach and successively re-attach, but not collectively detach to cause detachment of an entire bacterium, a computer simulation program was written using Labview (National Instruments, Austin, TX, USA), that uniquely advances existing simulation programs^29^ of tethered particle motion to include the dynamics of tether binding. The model is explained in more mathematical detail in the Supplementary Material, but its essentials are summarized here (see also Fig. S2). Briefly, an adhering (bio)particle experiences both deterministic and stochastic forces according to the Langevin equation^38^. The stochastic forces are generated by the thermal motion of the surrounding fluid molecules and result in Brownian-motion of adhering particles. Stochastic forces were accounted for as a white noise term added to the equation of motion^30^ that governs adhering particle motion in three dimensions, i.e. parallel and perpendicular to the substratum surface. Deterministic forces include the elastic forces that individual binding tethers exert on the surface after elongation or compression and the viscous drag from the surrounding fluid, while gravity and buoyancy forces were considered small enough to be neglected. Once the elastic force of an individual tether on the substratum surface exceeds its adhesion force, the program allows it to detach from its substratum position. Each tether that approaches the substratum surface closer than its length, is subsequently able to attach or re-attach. The model assumes spherically shaped particles, such as all bacterial strains used in this study, with 256 tethers equally distributed over the particle surface. Input parameters can be separated in particle-related and environmentally-related ones. Particle-related parameters include particle diameter and mass, the initial number of binding tethers per particle, their length, spring constant and individual tether adhesion force, while environmentally-related parameters are the surrounding fluid viscosity, density and temperature. Accordingly, position-maps can be generated *in silico* under different conditions that account for the dynamics of tether binding. In addition, the model yields the increase in the number of binding tethers over the experimental period and their residence times. The *in silico* generated position-maps can subsequently be analyzed in exactly the same way as experimentally observed position-maps.

## Electronic supplementary material

Supplementary Material

Supplementary Video
