# Supplementary material for: Detachment and successive re-attachment of multiple, reversibly-binding tethers result in irreversible bacterial adhesion to surfaces
Source: Sci Rep. 2017 Jun 29;7:4369. doi: 10.1038/s41598-017-04703-8 (PMC5491521; doi:10.1038/s41598-017-04703-8)
Supplement: Supplementary file 1 — Supplementary Material [file 41598_2017_4703_MOESM1_ESM.pdf]

SUPPLEMENTARY MATERIAL

Detachment and successive re-attachment of multiple, reversibly-binding tethers result in irreversible bacterial adhesion to surfaces

Jelmer Sjollema[1], Henny C. van der Mei[1]*, Connie L. Hall[2], Brandon W. Peterson[1], Joop de Vries[1], Lei Song[1], Ed D. de Jong[1], Henk J. Busscher[1] and Jan J.T.M. Swartjes[1]

[1] University of Groningen and University Medical Center Groningen, Department of Biomedical Engineering, Antonius Deusinglaan 1, 9713 AV , Groningen, The Netherlands.

[2]Department of Biomedical Engineering, The College of New Jersey, Armstong Hall, Room 181, P. O. Box 7718, The College of New Jersey, Ewing, NJ 08628, USA

Table S1 Physiological status and metabolic activity of the bacteria used during AFM and bacterial vibration spectroscopy, assessed by dead/live staining[1] and fluorescence microscopy[a] and metabolic activity determined with an MTT[2] (3-(4,5-dimethylthiazol-2-yl)-2,5-diphenyltetrazolium bromide) reduction colorimetric assay[b]. Data immediately after harvesting were identical to data obtained 2 h after harvesting, representing the maximum duration of either experiment. All experiments were carried out in triplicate with separately cultured bacteria. ± Signs indicate standard deviations.

BACTERIAL STRAIN	%LIVE BACTERIA (green fluorescent)		MTT ACTIVITY 5 x 10^8 bacteria per ml	
	During AFM	During VIBRATION SPECTROSCOPY	During AFM	During VIBRATION SPECTROSCOPY
Staphylococcus aureus ATCC12600	93 ± 3	90 ± 4	4.5 ± 0.6	3.2 ± 0.4
Staphylococcus aureus NCTC8325-4	97 ± 1	93 ± 2	4.3 ± 0.2	3.6 ± 0.3
Staphylococcus epidermidis ATCC35983	99 ± 1	94 ± 4	3.7 ± 0.4	2.8 ± 0.3
Staphylococcus epidermidis ATCC35984	95 ± 1	91 ± 4	4.5 ± 0.7	3.5 ± 0.1
Streptococcus mutans LT11	99 ± 1	93 ± 7	0.69 ± 0.08	0.26 ± 0.02
Streptococcus mutans IB03987	99 ± 1	96 ± 3	0.54 ± 0.10	0.26 ± 0.02
Streptococcus salivarius HB7	93 ± 4	92 ± 5	0.32 ± 0.09	0.30 ± 0.03
Streptococcus salivarius HBC-12	97 ± 2	97 ± 2	0.46 ± 0.18	0.42 ± 0.11

[a]each experiment involved enumeration of 100-150 live or dead bacteria.
[b]data represent absorbance at 560 nm.

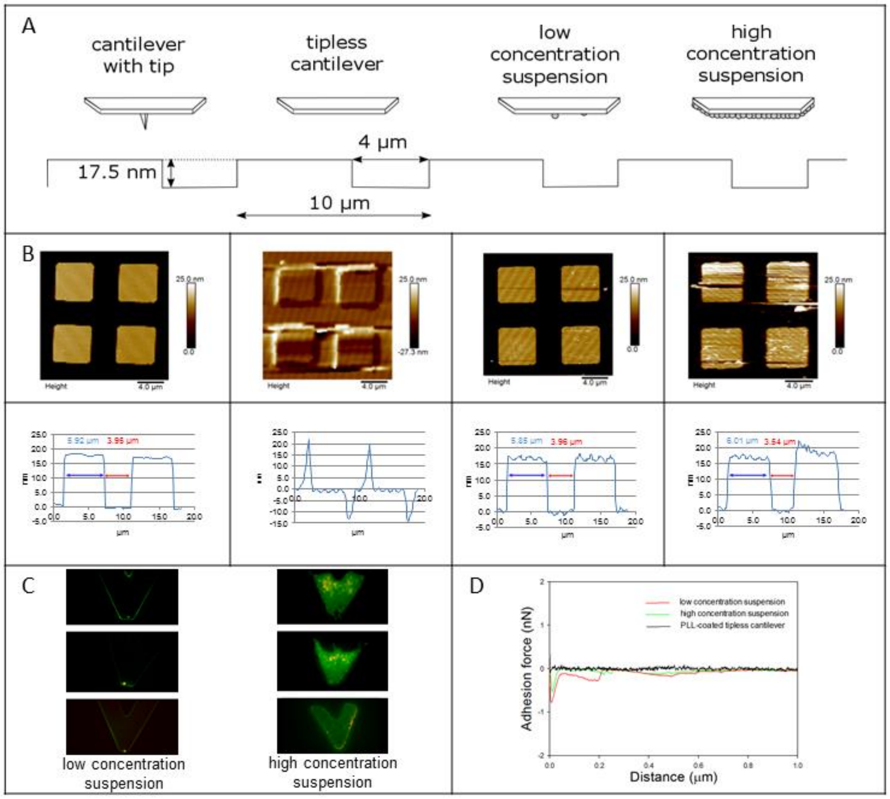

Figure S1. Demonstration of single-bacterium contact probe AFM.

(A) Schematic presentation of the topography of the calibration grid (HS-20MG BudgetSensors, Innovative Solutions Bulgaria Ltd., Sofia, Bulgaria) used to check whether bacterial AFM probes yield single-bacterium contact (grid depth not drawn to scale with the width of the wells). Multiple-bacterium contact of the AFM probe would not correctly reflect the dimensions of the grid and yield double contour lines in imaging.

(B) Image of the topography of the calibration grid (see panel A) obtained in the contact mode with
- a regular AFM tip (NP; Bruker AFM Probes, Camarillo, CA, USA; length and width at the top 3 and 1 µm respectively, with a half angle at the tip end of 18 degrees),
- a tipless, PLL-coated cantilever (NPO; Bruker; Camarillo, CA, USA) without bacteria attached and
- two cantilevers with bacteria (*S. salivarius* HB7) attached from a low (3 x 10^6 bacteria mL^{-1}) and high bacterial concentration (3 x 10^8 bacteria mL^{-1}) suspension. The image obtained with a tipless cantilever is completely vague, while especially images obtained with single-bacterium contact probe resemble the image obtained with the AFM tip. Note that no double contour lines are seen, indicative of single-bacterium contact. All bacterial probes yield the correct depth and width of the calibration grid, although the bacterial probe prepared from the high concentration suspension shows the smallest width and a minor distortion. The cantilever without attached bacteria does not reflect the grid dimensions at all. Increasing the loading force from 5 nN to 30 nN yielded similar images as obtained with both types of bacterial probes.

(C) Examples of fluorescence images of LIVE/DEAD stained *S. salivarius* HB7 (Baclight viability stain; Molecular Probes Europe BV, Leiden, The Netherlands) on tipless AFM cantilevers, prepared from a low and high concentration bacterial suspension. Viable bacteria are green fluorescent, red fluorescent

bacteria are membrane-damaged ("dead"). Images are presented for bacteria attached from low and high concentration suspensions (three cantilevers shown for each concentration).

(D) Force-distance curves *versus* glass measured with a PLL-coated cantilever without attached bacteria and with *S. salivarius* HB7 attached from a low and high concentration bacterial suspension.

***In silico* modeling of tethered particle motion with detaching and successively re-attaching tethers**

Tethered particle motion of an adhering (bio) particle in three dimensions is described in the Langevin equation as being governed by deterministic and stochastic forces. Deterministic forces include the viscous drag force arising from the surrounding fluid and elastic forces that individually attached tethers exert on a substratum surface after elongation or compression, while stochastic Brownian-motion forces are generated by the thermal motion of the surrounding fluid molecules. Accordingly

$$-\vec{F}_D - \vec{F}_{T,elastic} + \vec{F}_B = m\vec{a} \qquad \text{(S1)}$$

in which $\vec{F}_D$ is the viscous drag force, $\vec{F}_{T,elastic}$ the elastic force from the tether and $\vec{F}_B$ the Brownian-motion force, m is the particle mass and $\vec{a}$ is the resulting acceleration acting on the particle. Gravity and buoyancy forces were neglected. In our simulation program (see Fig. S2 for an overview), Brownian-motion forces for each time point i, after each time increment Δt, were computed as a white noise term from random numbers w_i, derived from a standard Gaussian distribution with mean of zero and standard deviation of unity[3,4]. Random numbers generated were scaled to the same level as the other forces appearing in Eq. S1 using

$$\frac{(F_B)_i}{m} = \sqrt{\frac{216\,\mu k_B T}{\pi C_c {\rho_p}^2 d_p^5 \Delta t}}\; w_i \qquad \text{(S2)}$$

in which

m	the particle mass (5.7×10^{-16} kg)
d_p	particle diameter (1000 nm)
μ	dynamic viscosity of the surrounding fluid (1×10^{-3} Pa s)
T	absolute temperature of the surrounding fluid (293 K)
C_c	Cunningham correction factor; set to 1 due to negligible slip in liquid media
k_B	Boltzmann constant (1.38×10^{-23} m^2 kg s^{-2} K^{-1})
Δt	Time step for each iteration (1×10^{-6} s)

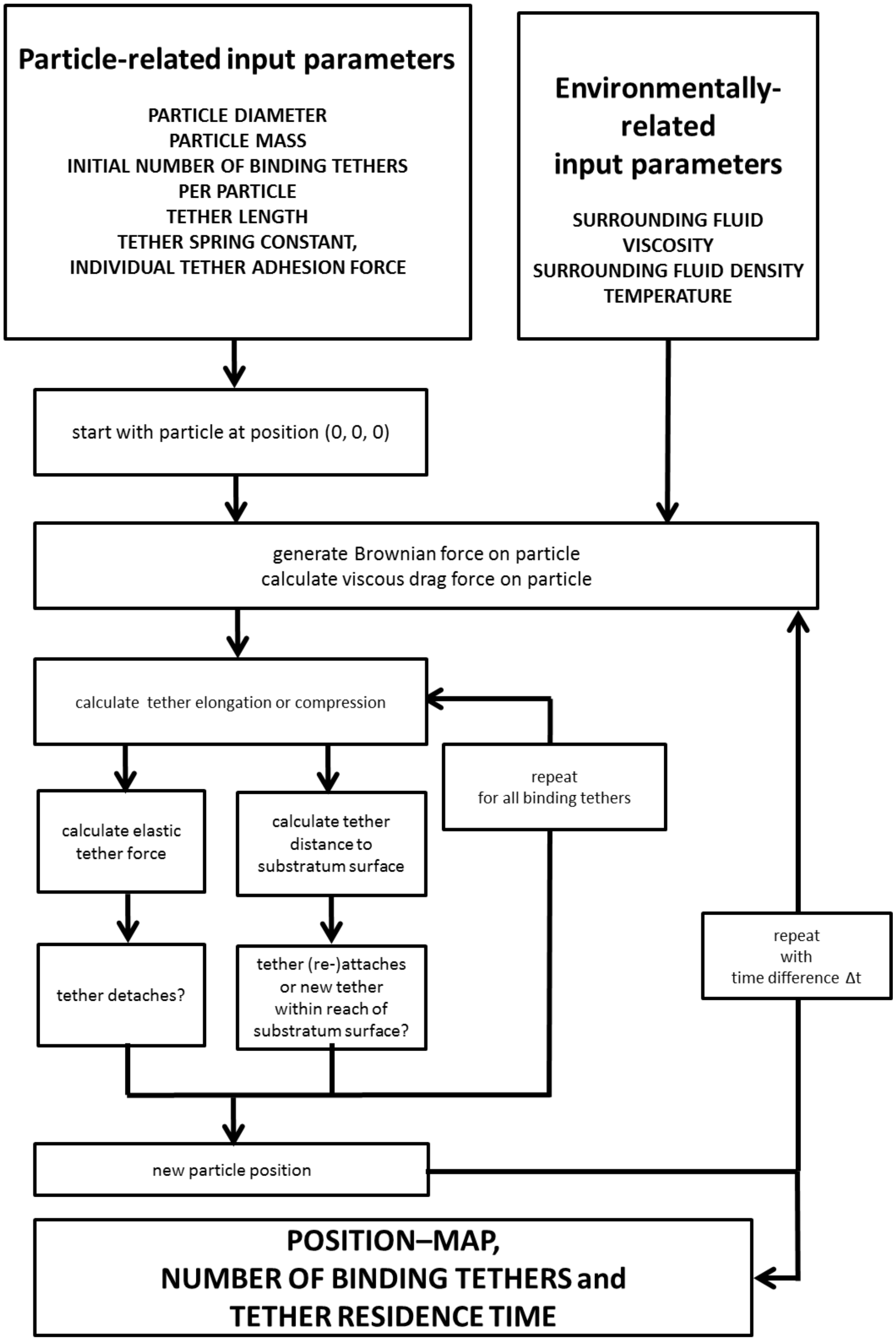

Figure S2. Schematics of the algorithm developed to simulate tethered particle motion accounting for detachment and re-attachment of binding tethers.

For the simulations in this paper, elastic forces from the tethers were calculated using a spring constant, k of 1.2 x 10^{-5} N/m, representing the average value over a variety of different bacterial strains and species measured using vibration spectroscopy[5]. Accordingly, elastic tether forces were computed based on the previous radial position ($\vec{r}_{i-1}$) of the center of the bacteria, assumed constant throughout the ith time step, Δt. Elastic forces of each individual binding tether were summed over all tethers

$$\left(\frac{\overrightarrow{\vec{F}_{T,elastic}}}{m}\right)_{i-1} = \sum_{j=1}^{N_t} \frac{k_j}{m} (ds_{i-1})\, \hat{I}_j \qquad \text{(S3)}$$

in which N_t is the number of binding tethers, ds the relative extension or compression of the tether, and $\hat{I}_j$ is the unit vector in the direction of the tether. For all simulations, 252 tethers were equally distributed over the particle surface, of which 12 were assumed to be involved in initial binding. In the current simulations, all tethers were taken of an equal length of 50 nm. Once the substratum surface came within reach of the tether length, the tether was allowed to bind to the substratum surface.

Viscous drag forces were accounted according to Stokes law

$$\left(\vec{F}_d\right)_{i-1} = 3\pi\mu d \frac{(d\vec{r}_{i-1} - d\vec{r}_{i-2})}{\Delta t} \qquad \text{(S4)}$$

in which $\vec{r}_i$ is the radial position of the center of an adhering particle.

Subsequently, a first and second order backward difference algorithm was implemented to calculate the location of the center of a particle, based its previous two positions. Initially, all tethers were oriented radially outwards as they would be in the planktonic phase prior to adhesion. Simulated particle adhesion and tethered particle motion was initiated by allowing the 12 initially binding tethers to adhere to the substratum surface and exposing the particle to the prevailing forces listed in Eq. S1. When the distance between the tether origin at the particle surface and the substratum surface was less than the tether length, taken as 50 nm within the current simulations, the tether was allowed to bind in addition to the 12 initial ones (of which a number may have detached within the process). During simulation, the elastic tether force was assumed to be radially directed in order to avoid particle torsion.

Tethers were detached in this simulation when the elastic force generated in an elongated tether exceeded the tether adhesion force, an input parameter to the program. Moreover, the probability, P_{det}, that a tether detached from the substratum surface was programmed to increase when the elastic force generated in the tether due to Brownian-motion and viscous drag forces increased according to

$$P_{det} = 10\% + (F_{T,\ elastic} - F_{T,\ adhesion}) \times 100\% \qquad \text{(S5)}$$

in which $F_{T,\ elastic}$ is the elastic tether force at the current position of the particle, i.e. the current elongation of the tether and $F_{T,\ adhesion}$ the individual tether adhesion force.

Accordingly, position-maps were generated in silico under different conditions that account for the dynamics of tether binding, including the final number of binding tethers and their residence times. The

in silico generated position-maps can subsequently be analyzed in exactly the same way as experimentally observed position-maps.

VIDEO V1. Time-lapse series of binding tethers of a simulated adhering particle initially binding with 12 tethers, each with a tether adhesion force of 0.18 pN. The dots indicate the positions of the binding tethers. Time step for each iteration in the simulation program was 1 x 10^{-6} s.

References

1. Alakomi, H. L., Matto, J., Virkajarvi, I. & Saarela, M. Application of a microplate scale fluorchrome staining assay for the assessment of viability of probiotic preparations. *J. Microbiol. Methods* **62,** 25-35 (2005).
2. Krom, B. P., Cohen, J. B., McElhaney Feser, G. E. & Cihlar, R. L. Optimized candidal biofilm microtiter assay. *J. Microbiol. Meth.* **68,** 421-423 (2007).
3. Li, A. & G. Ahmadi. Dispersion and deposition of spherical particles from point sources in a turbulent channel flow. *Aerosol Sci. Technol*. 1992. **16**, 209-226 (1992).
4. Volpe G, Volpe G. Simulation of a Brownian particle in an optical trap. *Am. J. Physics* 81: 224-230 (2013).
5. Song, L. *et al*. Nanoscopic vibrations of bacteria with different cell-wall properties adhering to surfaces under flow and static conditions. *ACS Nano* **8**, 8457–8467 (2014).
